# Older Female Farmers and Modeling of Occupational Hazards, Wellbeing, and Sleep-Related Problems on Musculoskeletal Pains

**DOI:** 10.3390/ijerph19127274

**Published:** 2022-06-14

**Authors:** Dong Seok Shin, Byung Yong Jeong

**Affiliations:** 1 Korea National Industrial Convergence Center, Korea Institute of Industrial Technology, Ansan 15588, Korea; dsshin@kitech.re.kr; 2 Department of Industrial and Management Engineering, Hansung University, Seoul 02876, Korea

**Keywords:** musculoskeletal disorders, occupational hazards, wellbeing, sleep-related problem, structural equation model

## Abstract

Musculoskeletal pains seem to be the most prevalent among occupational diseases in older female farmers. This study analyzes the relationships between exposure to ergonomic or environmental hazards, sleep-related problems, wellbeing, and musculoskeletal pains in older female farmers. In this study, older farmer means a farmer aged ≥60, and 2005 older female farmers were selected. A structural equation model (SEM) was used to investigate the relationships. In the results of SEM, musculoskeletal pains were more affected by the level of wellbeing (standardized path coefficient = −0.149) than the level of sleep-related problems (standardized path coefficient = 0.131) or the exposure level of ergonomic hazards (standardized path coefficient = 0.086). Among the ergonomic risk factors felt by elderly female farmers, the influence level of “awkward posture” (0.735), “repetitive motion” (0.718), or “standing posture” (0.693) was greater than that of “manual material handling” (0.661). “Vibration” (0.786) and “noise” (0.683) were influential variables of environmental hazards. In addition, “upper limb pain” (0.796) and “lower limb pain” (0.751) variables were more influential variables of musculoskeletal pains than the “backache” (0.472) variable. This study shows that strategies to improve wellbeing or sleep problems are important to alleviate or prevent musculoskeletal pains among older female farmers.

## 1. Introduction

Agriculture is a major source of food supply; however, the number of agricultural workers is declining because of long working hours, tough working conditions, and low wages [1]. The farm population of Korea decreased from 3.063 million people (6.0% of the entire Korean population) in 2010 to 2.215 million people (4.3% of the whole Korean population) in 2021 [1]. Furthermore, as there is no influx of young people, the aging phenomenon is intensifying in the agricultural population of Korea. The ratio of older farmers increased from 41.8% (1.279 million persons) in 2010 to 62.4% (1.381 million persons) in 2021 [1]. In this study, an older farmer means a farmer aged ≥60.

Despite advances in agricultural technology, agriculture is considered one of the sectors with many work-related injuries and illnesses [2]. Musculoskeletal pains seem to be the most prevalent among occupational diseases in farm households [3,4,5,6]. Agricultural work usually includes many physically demanding tasks that involve a combination of heavy material lifting, excessive force, and awkward posture [2]. Heavy material handling, hand stress caused by tools, repetitive gripping, and bending postures for a long time are related to upper limb pain [5,6]. Awkward or unsafe standing postures are also known to contribute to lower limb musculoskeletal disorders (MSDs) [7]. These ergonomic risk factors are important risks for agricultural workers, along with the risks posed by environmental factors (mainly working in cold and hot environments, as well as noise, vibration, and exposure to chemicals) [8]. In this study, the occupational hazard factors of farmworkers are classified into ergonomic hazards and environmental hazards. Uncomfortable posture, repetitive movements, and excessive force are known as ergonomic risk factors (ergonomic hazards). Environmental risk factors (environmental hazards) include vibration, noise, and skin contact with chemicals.

As women live longer than men, the proportion of the older population in female farmers is higher than that of males. The proportion of older female farmers increased from 43.8% (0.682 million persons) in 2010 to 63.4% (0.707 million persons) in 2021 [1,3]. Older farmers frequently have MSDs due to long-term exposure to occupational risk factors and workplace accidents [9,10]. Additionally, women have a higher risk of MSDs than men because they are more susceptible when lifting heavy objects and repetitive activities [11,12].

Older female farmers have problems of economic vulnerability, low education, and living alone [13]. Therefore, they have insufficient access to medical institutions, difficulty receiving appropriate treatment for chronic diseases, and a high risk of psychological problems [14]. Despite the feminization and living alone problems of older farmers, there are a lack of studies on the relationships between risk factors and musculoskeletal pains for older female farmers. Therefore, this study analyzes the relationship between exposure to work-related risk factors and older female farmers’ musculoskeletal pains.

In order to prevent musculoskeletal pains, it is important to investigate not only ergonomic or environmental working risk factors, but also the relationships between psychological factors [15,16,17]. Previous researchers have focused on the effects of sleep-related problems [18,19,20,21,22,23,24,25,26,27] and well-being (or depression) [27,28,29,30,31,32,33,34] as psychological factors related to musculoskeletal pains.

Physically strenuous farm work is associated with sleep-related problems [27]. Insomnia may contribute to the development of musculoskeletal pains [19]. In chronic pain, lack of sleep leads to higher pain the next day [21,24]. Therefore, this study investigates the effect of ergonomic or environmental risk factors on sleep-related problems and the impact of sleep-related problems on musculoskeletal pains in older female farmers.

Poor working conditions are associated with depression [30]. Depression (or wellbeing) is an important factor in increasing musculoskeletal pains [29,30,31,32]. However, the relationship is not known between MSD and wellbeing in older farmers. The WHO-5 index is useful for screening for wellbeing or depression [35,36]. Thus, this study investigates the effect of ergonomic or environmental risk factors on wellbeing and the impact of wellbeing on musculoskeletal pains in older female farmers.

Based on a literature review, this study expresses the relationship between work-related risk factors (including psychological factors) and musculoskeletal pains through seven hypotheses. That is, this study analyzes the effects of environmental hazards on wellbeing (Hypothesis 1) or sleep-related problems (Hypothesis 2). Second, this study investigates the effect of ergonomic hazards on wellbeing (Hypothesis 3) and sleep-related problems (Hypothesis 4). Third, this research analyzes the effect of exposure to ergonomic hazards on musculoskeletal pains (Hypothesis 5). Finally, this research examines the effect of sleep-related problems (Hypothesis 6) or wellbeing (Hypothesis 7) on musculoskeletal pains. This study used the structural equation model (SEM) to create a model merging seven interrelationships on musculoskeletal pains.

The aging and feminization phenomenon is intensifying in the agricultural population of Korea. Thus, creating a model merging the interrelationships of musculoskeletal pains is important to prevent musculoskeletal pains among older female farmers. This study aims to analyze the relationships between physical and psychological factors affecting musculoskeletal pains in older female farmers. That is, this study aims to verify the SEM model on musculoskeletal pains that combines the relationships between exposure to ergonomic hazards or environmental hazards, sleep-related problems, wellbeing, and musculoskeletal pains.

## 2. Methods

### 2.1. Data Collection and Subjects

This study was performed using the Fifth Korean Working Conditions Survey (KWCS) data set [37], which is similar to the European Working Conditions Survey (EWCS) [38]. Researchers can access the Fifth KWCS data [37], and this study used data sets and questionnaires. The Fifth KWCS was performed in accordance with the Korean Statistical Act, and this study used KWCS data in compliance with the research ethics.

Agriculture faces aging and feminization. This study focuses on the musculoskeletal pains of older female farmers, and an older farmer means a farmer aged 60 years old or older. That is, among the study variables, the gender variable was controlled as being female, and the age group was 60 years or older. Of the 59,205 participants in the KWCS survey, this study filtered out 2005 female farmers aged ≥60.

### 2.2. Research Variables

The variables of this study were selected from the items of the KWCS questionnaire [37] and the EWCS questionnaire [39]. The research variables consisted of latent variables related to ergonomic hazard exposure, environmental hazard exposure, sleep-related problem, wellbeing, and musculoskeletal pains.

Musculoskeletal pains was expressed as complaints of backache, upper limb pain, lower limb pain, or overall fatigue in the question of whether they had musculoskeletal pains in the last 12 months.

Farmer wellbeing was represented by the WHO-5 index [35,36]. The WHO-5 has five measurement variables representing feelings experienced over the last two weeks, namely: good spirits, relax, active, vitality, and interest [37,39]. Each measurement variable was expressed on a Likert scale from 1 to 5. Sleep-related problems were represented by the following three measurement variables: falling asleep, waking up, and feeling tired [37,39].

The ergonomic hazard exposure and environmental hazard exposure were evaluated as the level of exposure time to hazards and were categorized into ergonomic hazards and environmental hazards. Ergonomic hazards were composed of awkward posture, manual material handling (MMH), standing posture, and repetitive motion [37,39]. Environmental hazards were defined as physical and chemical hazards in the questionnaire [37,39], and consisted of vibration, noise, hot environment, cold environment, fumes and dust, vapors, skin contact with chemicals, and infection.

### 2.3. Data Analysis and Structural Equation Modelling

The hypotheses of this study are as follows:

**Hypothesis 1** **(H1).** *Environmental hazards affect wellbeing.*

**Hypothesis 2** **(H2).** *Environmental hazards affect sleep-related problems.*

**Hypothesis 3** **(H3).** *Ergonomic hazards affect wellbeing.*

**Hypothesis 4** **(H4).** *Ergonomic hazards affect sleep-related problems.*

**Hypothesis 5** **(H5).** *Ergonomic hazards affect musculoskeletal pains.*

**Hypothesis 6** **(H6).** *Sleep-related problems affect musculoskeletal pains.*

**Hypothesis 7** **(H7).** *Wellbeing affects musculoskeletal pains.*

This study combines the relationships between exposure to ergonomic hazards or environmental hazards, sleep-related problems, wellbeing, and musculoskeletal pains. Sleep-related problem and wellbeing were both an explanatory variable and a dependent variable in the model.

### 2.4. Reliability Analysis and Model Fit Test

The survey responses of older female agricultural workers were reliable if they had internal consistency in the response characteristics. In addition, the questionnaire survey was conducted with validity if it had the structural validity of being classified as the theoretically intended hazard factors. Reliability analysis was used to confirm the internal consistency of the measurement variables. The measurement variables for each latent variable were analyzed for internal consistency using Cronbach’s α value. In addition, a factor analysis through Varimax factor rotation was performed to assess the construct validity.

In this study, SEM was verified using the model fit test and composite reliability analysis. The model fit test was performed using the goodness of fit indices, such as χ2 and p values, normed fit index (NFI), goodness-of-fit index (GFI), and root mean square error of approximation (RMSEA) values. The composite reliability of the model was analyzed using the average variance extracted (AVE), composite reliability (CR), and correlation coefficients between variables. SPSS version 18.0 and AMOS 18 were used as the statistical analysis tools.

## 3. Results

### 3.1. Results of Reliability and Validity Analysis

Table 1 shows the results of the reliability analysis and factor analysis. The total standardized Cronbach’s α value was 0.756, indicating acceptable internal consistency. The factor analysis results were constructed of the following five component factors: (1) wellbeing, (2) ergonomic hazards, (3) sleep-related problems, (4) musculoskeletal pains, and (5) environmental hazards. The five factors were classified according to the factor analysis and internal consistency of questionnaire responses from older female workers. The Kaiser–Meyer–Olkin test (0.823 > criteria = 0.60) and Bartlett’s test (*p* < 0.001) were also significant. Thus, the variables and component factors showed satisfactory reliability and construct validity.

Although “hot” was an environmental hazard in the questionnaire, it was classified as an ergonomic hazard according to the internal consistency of responses to the questionnaires of older female workers. This indicates that the older female farmers responded to “hot” with a higher internal consistency for ergonomic hazards rather than environmental hazards.

### 3.2. Results of Model Fit and Hypothesis Testing

The results of the model fit tests were NFI = 0.928 (acceptable fit: 0.90 ≤ NFI), GFI = 0.932 (acceptable fit: 0.90 ≤ GFI), and RMSEA = 0.060 (acceptable fit: 0.05 < RMSEA ≤ 0.08). Therefore, they were evaluated as being an acceptable fit in the model fit results.

Table 2 shows the results of the convergent validity analysis using average variance extracted (AVE) and composite reliability (CR). In Table 2, the CR values were between 0.870 and 0.934 (acceptable criteria >0.70), so these results show a strong composite reliability. The AVE values were also greater than the correlations between the variables, so the results supported convergent validity.

Table 3 represents the results of the hypothesis testing for the proposed relationships. In Table 3, environmental hazards affected wellbeing (*p* = 0.014). In addition, environmental hazards had a significant impact on sleep-related problems (*p* < 0.001). Therefore, H1 and H2 were adopted statistically. However, Hypothesis 3 (H3: ergonomic hazards affect wellbeing) was not supported (*p* = 0.494). In contrast, ergonomic hazards affect sleep-related problems (*p* = 0.001). Thus, H4 was statistically supported. In addition, ergonomic hazards affect musculoskeletal pains (*p* = 0.002). Similarly, wellbeing (*p* < 0.001) or sleep-related problems (*p* < 0.001) have a significant effect on musculoskeletal pains. Therefore, H5, H6, and H7 were statistically supported.

### 3.3. Results of Structural Equation Modelling

**Figure 1:**
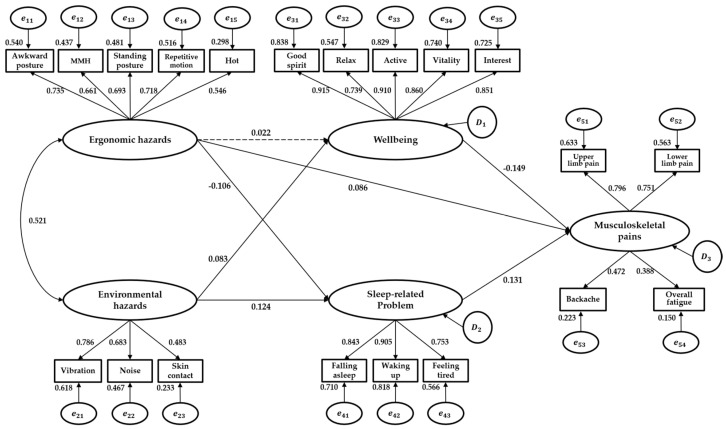
Final model of this study. Rectangles represent the measurement variable, and ellipses represent the latent variable. Di = disturbance or residual; ei = measurement error.

Figure 1 represents the final SEM. As shown in Figure 1, environmental hazards affected wellbeing (standardized path coefficient = 0.083). In addition, exposure to environmental hazards increased sleep-related problems (standardized path coefficient = 0.124). Exposure to ergonomic hazards decreased sleep-related problems (standardized pathway coefficient = −0.106). That is, sleep-related problems were more affected by the exposure level of environmental hazards (standardized path coefficient = 0.124) than the exposure level of ergonomic hazards (standardized path coefficient = −0.106). However, wellbeing was only affected by the exposure level of environmental hazards (standardized path coefficient = 0.083).

Exposure to ergonomic hazards increased musculoskeletal pains (standardized pathway coefficient = 0.086). In addition, sleep-related problems increased musculoskeletal pains (standardized path coefficient = 0.131). In comparison, wellbeing decreased musculoskeletal pains (standardized path coefficient = −0.149). That is, musculoskeletal pains were more affected by the level of wellbeing (standardized path coefficient = −0.149) than the level of sleep-related problems (standardized path coefficient = 0.131) or the exposure level of ergonomic hazards (standardized path coefficient = 0.086).

Awkward posture (0.735), repetitive motion (0.718), standing posture (0.693), and MMH (0.661) were influential variables of ergonomic hazards. Vibration (0.786) and noise (0.683) were influential variables of environmental hazards. All five variables of wellbeing were influential, and good spirit (0.915) and active (0.910) showed a strong influence. In addition, all three variables of sleep-related problems were influential, and wake up (0.905) had a strong influence. Upper limb pain (0.796) and lower limb pain (0.751) were influential variables of musculoskeletal pains.

## 4. Discussion

Agriculture faces a decline in employment and attractiveness compared with other sectors, with precarious working conditions and low income [12,13]. Thus, farm households are characterized by aging and feminization. In addition, as the number of people living alone increases, the psychological problems of farmers are being highlighted. This study analyzed the relationship between physical and psychological factors affecting musculoskeletal pains in older female farmers.

MSDs of farmers are caused by physical factors such as repetitive movements, awkward or stretched postures, and standing [4,8]. These ergonomic risk factors are important risks for agricultural workers, along with the risks posed by physical and environmental factors [4,8]. In this study, the influential ergonomic hazard variables felt by older female farmers were awkward posture, repetitive motion, standing posture, and MMH. Among the physical and environmental risk factors, vibration and noise were recognized as influential variables.

Musculoskeletal pains are common in elderly farmers [9]. The pain in the back and lower extremities of farmers increases with increasing workload, whereas the pain in the upper extremities is affected by MMH [40]. According to the results of this study, “upper limb pain” and “lower limb pain” variables were more influential variables of the musculoskeletal pains of older female farmers, unlike the low back pain that commonly occurs in farmers [41]. Moreover, among the ergonomic risk factors felt by elderly female farmers, the influence level of awkward posture (0.735), repetitive motion (0.718), or standing posture (0.693) was greater than that of MMH (0.661). This result explains the pain in the upper and lower extremities of elderly female farmers. Female farmers have much pain in the upper extremities and lower extremities because many types of work, such as planting rice and seedlings, standing for a long time, and moving their hands repeatedly [4,42]. Older, experienced farmers either perform less strenuous physical work [3,41] or complete their tasks in a way that reduces pain, discomfort, and injuries [5,43]. In addition, this is partially consistent with previous results, that upper extremity pain is the most frequent musculoskeletal pain in farmers [5,43], and uncomfortable or unstable standing posture causes lower extremity pain [7].

Gender differences exist in physical vulnerability or sensitivity to pain, and women complain of more frequent and severe musculoskeletal pains than men [11,12,40,44,45]. Most older female farmers have problems with economic openness, low education, living alone, and difficulty receiving appropriate treatment for chronic diseases [13,14]. Although they have experienced musculoskeletal injuries, they are forced to continue to work. They tend not to visit medical facilities, and MSDs can be exacerbated [45]. Many do not consider their symptoms to be severe until they cannot perform specific tasks [41]. Sustainable work requires supporting living and working conditions that enable older people to continue to work longer [46]. The challenge is to match an individual’s needs and abilities with the quality of jobs on offer [46]. Interventions should reduce risk factors by improving working conditions [2,43]. That is, strategies are needed to adapt working conditions to suit older workers with decreased physical abilities [47,48]. Ergonomic interventions support the agricultural workforce by examining how workers prevent excessive force, repetitive motions, and awkward postures by improving tools and workplaces [2]. They can also prevent health problems by reducing exposure to environmental hazards for older workers [49].

The mechanization of agricultural work reduces the arduous work associated with MSDs. Although mechanization has reduced farmers’ work and has made their work more sustainable, at the same time, machines can also be a major risk factor due to a lack of safety mechanisms, high noise, and vibration [50]. Improving the working conditions of older farmers requires interventions that consider the ergonomics of older female farmers when designing agricultural equipment or systems [51].

The growth of new technologies changes the working environment, so training is needed in order to improve the skills of older workers. A well-designed training method that considers the characteristics of work and workers is effective for older workers [10,52]. It is important to train workers in a participatory approach with ergonomic knowledge. In addition, the prevention of risk is desirable as an interdisciplinary action considering environmental and process aspects, socio-cultural dynamics, and user-centered design [53].

In this study, the musculoskeletal pains of elderly female farmers were more affected by the level of wellbeing and sleep-related problems than the level of exposure to ergonomic hazards. This study suggests that strategies to improve wellbeing or sleep problems are important to alleviate or prevent musculoskeletal pains among older female farmers. Additionally, depressive symptoms (a negative conception of wellbeing), sleep problems, and musculoskeletal pains may be associated with an increased risk of experiencing injuries on farms [54]. By identifying the three symptoms and treating the related disorders, farmers can reduce occupational injuries. Therefore, it is important to identify and effectively treat depression, sleep disturbance, and musculoskeletal pains among farmers [27]. It is necessary to understand psychological symptoms such as sleep-related problems and wellbeing. Health promotion activities [55] are needed to enhance the impact of organizational factors [56] or psychosocial factors [57] in the workplace. Health promotion programs to improve workers’ mental health may be another way to improve their quality of life [58]. In addition, well-designed interventions to improve sleep-related problems might be potentially modifiable factors as part of the treatment for musculoskeletal pains [26,59]. Cognitive-behavioral therapy can effectively reduce sleep-related problems and positively affect musculoskeletal pains in the elderly [60].

This study has some limitations. First, the respondent’s musculoskeletal pains may be subjective. As this study only considered the presence or absence of subjective pain for musculoskeletal symptoms, it does not represent MSDs by actual diagnosis. In addition, the limitations (subjective musculoskeletal pains) should be expanded substantially to include other limitations related to each variable category, as well as how the variables were captured (e.g., recall bias on most variables, accuracy of assessing ergonomic, and environmental hazards). Second, the education, cognitive function, and habitual physical activity of the older female farmers were not assed in this study, and these important shortcomings are limiting factors of this study. Third, as this study was analyzed based on female farmers aged ≥60, the generalization of the results requires attention. For example, although “hot” is an environmental hazard in the questionnaire, it is classified as an ergonomic factor according to the internal consistency of older female farmers. Thus, further research is needed by expanding gender and age groups to overcome these limitations. Despite these limitations, this study showed that strategies to improve wellbeing or sleep problems are important to alleviate or prevent musculoskeletal pains among older female farmers.

## 5. Conclusions

This study shows that older female farmers have a high frequency of musculoskeletal pains in the upper and lower extremities. Additionally, this study demonstrates the importance of supporting older female farmers for addressing well-being or sleep problems in order to alleviate or prevent musculoskeletal pains.

## Figures and Tables

**Table 1:** Results of the factor analysis and reliability analysis.

Latent Variable	Cronbach’s α	Measurement Variable	Component				
			1	2	3	4	5
Wellbeing	0.931	Good spirit	0.926	0.025	0.017	−0.021	0.051
		Active	0.920	0.029	0.024	−0.044	−0.004
		Vitality	0.890	0.057	−0.031	−0.062	0.042
		Interest	0.867	0.000	0.057	−0.123	0.046
		Relax	0.812	−0.025	−0.044	0.074	0.006
Ergonomic hazards	0.800	Repetitive motion	−0.027	0.808	−0.113	0.060	0.031
		Awkward posture	0.092	0.792	0.019	0.202	0.074
		Standing posture	0.025	0.766	−0.020	−0.072	0.127
		MMH	0.028	0.660	0.026	−0.041	0.334
		Hot *	−0.032	0.592	0.037	0.012	0.253
Sleep-related problems	0.871	Falling asleep	−0.014	−0.039	0.904	0.109	0.026
		Waking up	−0.018	−0.044	0.890	0.053	0.041
		Feeling tired	0.042	0.028	0.862	0.054	0.045
Musculoskeletal pains	0.691	Upper limb pain	−0.073	0.035	−0.010	0.812	0.010
		Lower limb pain	−0.124	−0.022	−0.027	0.800	−0.006
		Backache	0.039	−0.040	0.113	0.665	−0.069
		Overall fatigue	0.009	0.146	0.112	0.566	−0.078
Environmental hazards	0.671	Noise	0.029	0.203	−0.022	−0.029	0.812
		Vibration	0.023	0.162	0.118	−0.182	0.777
		Skin contact	0.057	0.219	0.020	0.028	0.627
Instrument Total	0.756	% of Variance	20.47%	16.63%	12.91%	10.5%	5.63%
		Cumulative (%)	66.177				
		Kaiser-Meyer-Olkin Test	0.823				
		Bartlett’s Test	*p* < 0.001				

* Although it is an environmental hazard in the questionnaire, it is classified as an ergonomic factor according to internal consistency.

**Table 2:** Results of the composite reliability analysis.

Hypothesis	Ergonomic Hazards	Wellbeing	Sleep-Related Problem	Musculoskeletal Pains	AVE	CR
Environmental hazards	0.526	0.095	0.077	−0.156	0.831	0.934
Ergonomic hazards		0.066	−0.042	0.093	0.575	0.870
Wellbeing			0.004	−0.143	0.617	0.889
Sleep-related problem				0.130	0.700	0.874
Musculoskeletal pains					0.728	0.908

AVE = average variance extracted; CR = composite reliability.

**Table 3:** Results of hypothesis testing for the proposed relationships.

Hypothesis	Paths	Standardized Coefficient *(r)*	Critical Ratio	*p*-Value	Result
H1	Environmental hazards →Wellbeing	0.083	2.462	0.014 *	Supported
H2	Environmental hazards →Sleep-related problem	0.124	3.519	<0.001 *	Supported
H3	Ergonomic hazards →Wellbeing	0.022	0.684	0.494	Not Supported
H4	Ergonomic hazards →Sleep-related problem	−0.106	−3.179	0.001 *	Supported
H5	Ergonomic hazards →Musculoskeletal pains	0.086	3.085	0.002 *	Supported
H6	Wellbeing →Musculoskeletal pains	−0.149	−5.586	<0.001 *	Supported
H7	Sleep-related problem →Musculoskeletal pains	0.131	4.817	<0.001 *	Supported

* Significant difference at 0.05.
